# The relation between parental chronic pain, pain‐related attention and interpretation biases in pain‐free adolescents

**DOI:** 10.1002/ejp.1444

**Published:** 2019-07-15

**Authors:** Jantine J. L. M. Boselie, Mariëlle E. J. B. Goossens, Peter Muris, Linda M. G. Vancleef

**Affiliations:** ^1^ Clinical Psychological Science Maastricht University Maastricht The Netherlands; ^2^ Rehabilitation Reseach & Clinical Psychological Sciences Maastricht University Maastricht The Netherlands

## Abstract

**Background:**

Children of chronic pain patients run greater risk for developing chronic pain themselves. Exposure to chronic pain of the parent might install cognitive (e.g., pain catastrophizing, interpretation and attentional bias) and affective (e.g., pain anxiety) vulnerability which increase the risk for the development of chronic pain complaints in offspring. This study examines whether pain‐free offspring of parents with chronic pain complaints make more health‐threatening interpretations and display a stronger pain‐related attentional bias compared to the offspring of pain‐free parents. We furthermore examined differences between both groups on pain catastrophizing, pain anxiety and somatic symptoms and explored the relations between parental pain catastrophizing and aforementioned pain vulnerability measures in offspring.

**Methods:**

Offspring of parents with chronic pain complaints (*n* = 24) and pain‐free parents (*n* = 27) completed measures of attentional bias (i.e., pictorial dot probe), interpretation bias (i.e., ambiguous word association task), pain catastrophizing, pain anxiety and somatic symptoms. Parents completed measures of pain catastrophizing and psychological distress.

**Results:**

No differences between offspring of parents with and without pain complaints were observed on pain catastrophizing, pain anxiety and somatic symptoms. Both groups of healthy adolescents predominantly showed benign, non‐health‐threatening interpretations. Children of pain‐free parents showed an attention bias for pain stimuli, while offspring of parents with pain complaints showed no such bias.

**Conclusions:**

Future research is needed to further elucidate the precise role of parental pain in the development of pain‐related biases and the significance of these biases in the onset and/or maintenance of a chronic pain condition in children and adolescents.

**Significance:**

Parental chronic pain may install psychological vulnerability for developing chronic pain and associated complaints in offspring. This study did not show differences in pain‐directed attentional and interpretation bias between offspring of parents with chronic pain complaints and offspring of pain‐free parents. Further (longitudinal) research is needed to elucidate the precise role of parental pain factors in the development of pain‐related vulnerability in offspring of chronic pain parents, thereby identifying important targets for the prevention and early intervention of chronic pain.

## 1 INTRODUCTION

Chronic pain constitutes a major health care problem in the Western world, with almost 1 out of 5 adult Europeans suffering from this disabling condition (Breivik, Collett, Ventafridda, Cohen, & Gallacher, 2006). Children of chronic pain patients run greater risk for developing the disorder (Zadro et al., 2018), with 12%–62% of the adolescents with chronic pain having a parent who also suffers from this condition (Higgins et al., 2015; Stommen, Verbunt, Gorter, & Goossens, 2012)*.* Interpersonal models of pain indeed advocate the role of parental influences on children's pain and associated psychological outcomes (e.g., Palermo, Valrie, & Karlson, 2014; Simons & Kaczynski, 2012). Recently, Stone and Wilson (2016) proposed an integrative conceptual model for the transmission of risk for chronic pain from parents to their children. A key element of this model is that social learning mechanisms, including modelling and reinforcement of maladaptive child pain responses, promote the installation of cognitive and affective vulnerability for pain in offspring (Bandura and Walters 1977; Evans et al., 2008; Stone, Bruehl, Smith, Garber, & Walker, 2018).

Cognitive factors, in particular cognitive biases (i.e., the tendency to prioritize processing of information that is in accordance with the present concerns of an individual), are thought to play an important role in the onset and maintenance of chronic pain (Stone et al., 2018; Stone & Wilson, 2016). Both attentional (i.e., the selective allocation of attention towards pain‐related stimuli) and interpretation (i.e., the tendency to interpret ambiguous stimuli in a pain‐related/health‐threatening manner) biases have been linked to poor pain outcomes, both in adult (Crombez, Ryckeghem, Eccleston, & Damme, 2013; Pincus & Morley, 2001; Schoth & Liossi, 2016) and paediatric pain populations (Beck et al., 2011; Boyer et al., 2005; Heathcote, Jacobs, Eccleston, Fox, & Lau, 2017; Heathcote et al., 2016; Lau et al., 2018; van der Veek et al., 2014).

Prior research has shown that cognitive biases are promoted by parents in offspring for other psychological disorders, such as anxiety disorders, depression, insomnia and addictive behaviours (Dearing & Gotlib, 2009; Ellis, Thomson, Gregory, & Sterr, 2013; Forestell, Dickter, Wright, & Young, 2012; Kirsten, Roy, Hubert, & Rutger, 2012; Muris, Zwol, Huijding, & Mayer, 2010). In a similar way, parental pain might enhance attention to pain and threat interpretations of pain in offspring (Stone & Wilson, 2016). Besides aforementioned cognitive biases, other cognitive (i.e., pain catastrophizing, health beliefs) and affective (pain anxiety) factors are also assumed to play an important role in the developmental psychopathology of chronic pain (Asmundson, Wright, & Hadjistavropoulos, 2000; Vervoort, Eccleston, Goubert, Buysse, & Crombez, 2010).

The current study examined whether pain‐free offspring of parents with chronic pain complaints show more pronounced pain‐directed attentional and interpretation biases compared to offspring of pain‐free parents. Further, we investigated whether both offspring groups differ on pain catastrophizing, pain anxiety and somatic symptoms. Finally, given recent findings that parental pain coping behaviours, rather than the pain status per se are important for the transmission of the risk of pain from parents to offspring (Cordts, Stone, Beveridge, Wilson, & Noel, 2019; Simons & Kaczynski, 2012; Stone et al., 2018; Stone & Wilson, 2016), we also explored whether elevated levels of parental pain catastrophizing and parental psychological distress (i.e., anxiety, depression) are associated with increased cognitive and affective pain vulnerability in the offspring.

## 2 METHODS

### 2.1 Participants

A total of 51 pain‐free adolescents (*M* = 14.22 years, *SD* = 1.78, range 12–17 years; 28 boys) and their parents (*M* = 45.73 years, *SD* = 3.69; 8 males) participated in the study. Participants were allocated in two groups: those with and those without a parent suffering from chronic pain complaints (i.e., pain duration >6 months).

The parental pain group consisted of 24 adolescents (*M* = 14.42 years, *SD* = 1.79, 14 boys) and their parents (*M* = 45.88 years, *SD* = 3.44; 3 males). All but 1 parent reported to have formally received a chronic pain diagnosis (95.8%). The mean pain duration of parents in the chronic pain group was 13.8 years (*SD* = 10.93),^1^ with 17 (70.8%) parents reporting pain in multiple parts of their body and 12 (50%) parents reporting to also suffer from diseases, such as arthrosis, asthma, allergies, thyroid dysfunction, migraine and hernia. The pain‐free control group consisted of 27 adolescents (*M* = 14.04 years, *SD* = 1.79; 14 boys) and their parents (*M* = 45.59 years, *SD* = 3.96; 5 males). Information about work status, and relationship status of participating parents and school absenteeism of participating adolescents is presented in supplementary materials (Table S1).

Participants were recruited via primary and secondary schools, patient associations and database clinic of adult chronic pain patients who had previously indicated to be willing to participate in research. Inclusion criteria for the chronic pain group were as follows: (a) at least one of the parents reported to suffer from any form of musculoskeletal chronic pain and this parent was willing to participate in the study; and (b) the chronic pain complaints were present for at least 6 months. The main inclusion criterion for adolescents in the control group was that their parents did not suffer from chronic pain complaints or any other serious medical condition. For both groups other inclusion criteria were: (a) the child had no pain condition; (b) the child was between 12 and 17 years old; (c) the child lived together with the parent; (d) the child did not suffer from dyslexia and both parent and child had a good comprehension of the Dutch language; and (e) the parent and child did not suffer from psychological disorders. The Ethical Research Committee of the Faculty of Psychology and Neuroscience at Maastricht University approved the study protocol.

### 2.2 Questionnaires adolescents

#### 2.2.1 Pain catastrophizing

The Dutch Pain Catastrophizing Scale for Children (PCS‐C; Crombez et al., 2003) was used to measure the level of pain catastrophizing. The PCS‐C consists of 13 items that reflect catastrophic thoughts and emotions regarding pain (e.g., “When I have pain, there is nothing I can do to reduce the pain”). Adolescents indicate to what degree each of 13 items apply to them, using a 5‐point Likert scale ranging from 0 (“not at all”) to 4 (“extremely”). The total score ranges from 0 to 52, with higher scores indicating greater pain catastrophizing. The PCS‐C has been shown to be both reliable and valid (Crombez et al., 2003). In this study, the internal consistency was good, with a Cronbach's alpha of 0.84.

#### 2.2.2 Anxiety sensitivity

The childhood anxiety sensitivity index (CASI) (Silverman, Fleisig, Rabian, & Peterson, 1991) was used to measure the extent to which the child believes that the experience of anxiety will result in negative consequences. The CASI consists of 18 items (e.g., “It scares me when my heart beats fast”), that are rated on a 3‐point Likert scale ranging from 1 to 3 (“none,” “some” or “a lot”). The total CASI score can be obtained by summing the responses of all items (range 18–54) with higher scores reflecting greater anxiety sensitivity. The CASI has been shown to be a reliable and valid scale (Silverman et al., 1991). In the current study, the internal consistency was acceptable, with a Cronbach's alpha of 0.78.

#### 2.2.3 Somatic symptoms

The Dutch version of the children's somatization inventory (CSI; now known as children's somatic symptoms inventory) (Meesters, Muris, Ghys, Reumerman, & Rooijmans, 2003; Stone et al., 2019; Walker, Beck, Garber, & Lambert, 2008; Walker and Garber 2018) was used to measure the intensity of somatic symptoms as experienced in the past 2 weeks. The CSI contains 35 items (e.g., “How much were you bothered by lower back pain”) that are scored on a 5‐point scale ranging from 0 to 4 (“not at all,” “a little,” “somewhat,” “a lot” and “a whole lot”). A total score (range 0–140) is computed by summing the scores across all items, with higher scores indicating a higher intensity of somatic symptoms. The CSI has been shown to be reliable (Meesters et al., 2003) and in the present study the internal consistency was also good, with a Cronbach's alpha of 0.86.

### 2.3 Questionnaires parents

#### 2.3.1 Pain Catastrophizing

The Dutch translation of the Pain Catastrophizing Scale (PCS; Sullivan, Bishop, & Pivik, 1995) consists of 13 items reflecting catastrophic thoughts and emotions regarding pain (e.g., “I keep thinking about how much it hurts”). Scoring is similar to that of the PCS‐C (see above). Reliability and validity of the PCS total and subscales are adequate (Severeijns, Hout, Vlaeyen, & Picavet, 2002). In this study, internal consistency was good, with a Cronbach's alpha of 0.90.

#### 2.3.2 Depression and anxiety

The Hospital Anxiety and Depression Scale (HADS; Zigmond & Snaith, 1983) contains 14 items of which 7 measure depressive symptoms (e.g., “I feel as if I am slowed down”) and 7 assess anxiety symptoms (e.g., “I get a sort of frightened feeling as if something awful is about to happen”). Items are rated on a 4‐point Likert scale ranging from 0 (no symptoms) to 3 (high level of symptoms). The total depression or anxiety score can be calculated by summing scores on relevant items (after reversing scores on positively phrased items). The HADS has been demonstrated to be a reliable self‐report instrument (Spinhoven et al., 1997). Cronbach's alpha for the total scale was good (0.83), whereas coefficients for the anxiety and depression subscale were sufficient (0.77 and 0.74, respectively).

### 2.4 Cognitive bias measures

#### 2.4.1 Attention bias

A pictorial dot‐probe task was used to measure attentional bias towards pain‐related facial stimuli (Broeren, Muris, Bouwmeester, Field, & Voerman, 2011). The probe‐classification version required participants to identify the type of probe and was presented on a Dell laptop and programmed in E‐prime 2.0 (Psychology Software Tools Inc.). The facial stimuli were selected from the STOIC‐database (Roy et al., 2007). Only pictures of which the positive indication value was close to 1.00 were included in the study (an indication value of 0.96 for a picture displaying pain signified that in 96% of the cases, the picture was recognized as the emotion pain). In total, 20 pictures of 8 actor models (4 male and 4 female) were selected. Each model displayed 3 facial expressions: pain, happy and neutral. For each model, 16 trials were administered in which a neutral face picture was always paired with either a pain or a happy face picture. The location (i.e., left or right) of the emotional picture (i.e., pain or happy), the location of the probe (i.e., left or right) and probe type (i.e., one dot or two dots) were counterbalanced, resulting in 128 trials emotion‐neutral pairs. There were also 32 trials, where a neutral face picture was paired with a neutral face picture. Consequently, the dot‐probe task consisted of 160 trials in total. The test phase started with 8 practice trials, which contained only neutral faces that were not used during the testing phase. A trial started with a fixation cross presented for 500 ms in the centre of the computer screen. The fixation cross was followed by a pair of facial pictures (pain/neutral, happy/neutral or neutral/neutral), with one picture appearing on the left and the other picture on the right side of the screen for 500 ms. Then, the pictures disappeared and a probe, which was either one dot (“.”) or two dots (“..”) was immediately presented on the location of one of the two displayed faces. Adolescents were asked to press one of two response keys on a QWERTY keyboard (“Q” = “.”; “P” = “..”) to indicate the type of probe as quickly as possible while avoiding mistakes. Responses were provided using the index finger of the right and left hand. The probe was shown until a response was made. Reaction times (RTs) in ms were recorded. An attention bias score (Baum, Schneider, Keogh, & Lautenbacher, 2013) was calculated for each type of emotional expression (i.e., pain and happy) by subtracting the average reaction time on congruent trials (i.e., trials in which the pain or happy picture was replaced by the probe) from the average reaction time on incongruent trials (i.e., trials in which the neutral picture was replaced by the probe).

#### 2.4.2 Interpretation bias

A word association task was developed for the purpose of this study to assess a health‐threatening interpretation bias in adolescents. In this task, Dutch homographs (i.e., words with double meanings) were displayed in the middle of the page in an A5‐booklet. Adolescents were instructed to read each word and to write down the first word that came to mind when reading this word. They were instructed to create words as fast as they could and were prohibited to return to prior pages. They could proceed with the word on the next page only when the current page had been completed. In total, the task consisted of 14 homographs, with 7 health‐related (e.g., needle; critical homograph) and 7 non‐health‐related homographs (e.g., surfing; neutral fillers). These homographs were selected based on a pilot study with adolescents aged between 12 and 17 years (*N* = 39; 14 boys). For a list of 16 homographs, accompanied by one health‐related word and one non‐health‐related word, they were asked to indicate (in a percentage score 0%–100%) how much they thought the (i.e., health‐ or non‐health‐related) words were related to the homographs. Homographs were considered eligible to be incorporated in the task if the rating for both the health‐ and non‐health‐related word were both around 50%. Homographs for which one of the ratings exceeded 35% or 65% association ratings were discarded.

Participants were randomly assigned to complete an A or B version of the word association task. Both versions contained the same set of 14 homographs but differed regarding the presentation order of the words. The responses to the seven critical homographs (i.e., the words created by the participants) were subsequently categorized by two independent raters as either “health‐related” or “non‐health‐related.” The proportion of health‐related and non‐health‐related words was then calculated as the amount of words belonging to each category divided by the total amount of critical homographs (*n* = 7). An interpretation bias index was calculated as the difference score that resulted from subtracting the proportion of non‐health‐related words from the proportion health‐related words.

#### 2.4.3 Procedure

In/exclusion criteria were first checked via a telephone interview with the parent. Next, a test session was scheduled with the experimenter, during which both the parent and the adolescent completed all measures. Depending on the preference of participants, testing took place in their home environment or in an office at the university. After providing informed consent, the parent provided information concerning sociodemographic characteristics of themselves (i.e., age, sex, employment, presence and duration of pain complaints and relationship status) and the child (i.e., age, sex, school absence and health/pain status). Next, the parent completed the PCS and HADS and was asked to wait in silence until the child was finished. The adolescents first gave informed consent, after which he/she completed the dot‐probe task followed by the word association task and the questionnaires (i.e., PCS‐C, CASI and CSI). At the end of the session, the adolescents and the parent were debriefed and compensated for their combined contribution by means of a gift voucher.

#### 2.4.4 Data reduction and statistical analyses

First, the distribution of the data was checked and participant characteristics and internal consistency of the questionnaires were calculated. Data analysis of the dot probe was only conducted on correct trials (96.5% of all trials). Data of 1 participant had to be discarded due to high error percentage (47%). Reaction times (RT) below 250 ms were considered as anticipatory responses and removed from the data set. We adopted a rescaling method to reduce the influence of individual outliers on the reaction time scores while still maintaining all data points. Outliers were reassigned to a value of 3*SD* above individual mean RT such that they were within the normal range (1.5%; Price et al., 2015).

Independent samples *t* tests were used to check for baseline differences in parent pain catastrophizing and parent psychological distress between the chronic pain complaints and the pain‐free group. Next, a series of one‐way ANCOVA’s were run to examine group differences in attentional bias (for happy and painful faces) and interpretation bias scores between healthy offspring from parents with and without chronic pain complaints. The presence of attentional and interpretation biases within each group was subsequently examined with one sample *t* tests. Differences in pain catastrophizing, anxiety sensitivity and somatic symptoms were also examined by means of one‐way ANCOVA’s. Adolescent age was entered as a covariate in all ANCOVA’s to control for age effects given that the prevalence of chronic pain has been shown to increase with age (Perquin et al., 2000). The relations between parental pain catastrophizing, parental psychological distress and pain vulnerability outcomes in the offspring (attentional and interpretation biases, pain catastrophizing, anxiety sensitivity, somatic symptoms) were explored with Pearson correlation coefficients. All tests were performed at significance level of *α* = 0.05 and effect sizes (Cohen's d, partial eta squared) were presented as indication of strength of the effects.

## 3 RESULTS

### 3.1 Parents

#### 3.1.1 Pain catastrophizing, anxiety and depression

Results of the independent samples *t* tests showed that parents with chronic pain complaints showed more pain catastrophizing than pain‐free parents (Table 1). No group differences were observed with respect to anxiety and depression scores.

**Table 1:** Mean scores on pain catastrophizing, anxiety and depression scales for both groups of parents

	Parents with chronic pain complaints (*n* = 24)		Pain‐free parents (*n* = 27)		*t*	*p*	*d*
	*M* (*SD*)	range	*M* (*SD*)	range			
Pain catastrophizing	13.88 (9.03)	0–34	7.89 (6.28)	0–24	2.774	0.01	0.79
HADS total	9.08 (4.84)	1–20	8.48 (5.77)	1–25	0.400	0.69	0.11
Anxiety subscale	5.13 (2.95)	0–12	4.74 (3.38)	1–13	0.430	0.67	0.12
Depression subscale	3.96 (2.76)	0–10	3.74 (3.11)	0–12	0.263	0.79	0.08

### 3.2 Children

#### 3.2.1 Attentional bias

An ANCOVA revealed no difference in attentional bias scores for pain or happy faces between offspring from parents with and without chronic pain complaints (see Table 2). The covariate adolescent age was not significant (all *p *> 0.41). Results of the one‐sample *t* test revealed that the offspring of pain‐free parents showed a significant attention bias to painful faces, with *t*(26) = 2.27, *p *= 0.03, 95% CI [1.40–27.99], but not towards happy faces, with *t*(26) = 0.66, *p *= 0.52, 95% CI [−8.29 to 16.13]. Offspring of parents with chronic pain complaints did not show an attentional bias towards pain (*t*(22) = 1.16, *p *= 0.26, 95% CI [−5.62 to 19.90]) nor happy faces (*t*(22) = 0.03, *p *= 0.98, 95% CI [−13.45 to 13.06]). The attention bias measures did not correlate with parental pain catastrophizing (pain *r *= 0.13, *p *= 0.37; happy *r *= −0.01, *p *= 0.94), parental anxiety (pain *r *= −0.08, *p* = 0.57; happy *r *= −0.06, *p *= 0.66) or parental depression level (pain *r *= 0.02, *p *= 0.87; happy *r *= 0.08, *p *= 0.60).

**Table 2:** Mean attentional and interpretational bias scores for adolescents with and without a parent with chronic pain complaints

	Offspring of parents with chronic pain complaints *n* = 23	Offspring of pain‐free parents *n* = 27	*F*	*p*	η_p_^2^
	*M* (*SD*)	*M* (*SD*)			
Attentional bias					
Pain faces	7.14 (29.51)	14.69 (33.61)	0.818	0.37	0.02
Happy faces	−0.19 (30.66)	3.92 (30.86)	0.159	0.69	0.00
Interpretation bias	−42.26 (34.60)	−52.91 (30.64)	0.868	0.36	0.02

#### 3.2.2 Interpretation Bias

Results of the ANCOVA on the interpretation bias index showed no significant difference between offspring of parents with chronic pain complaints and offspring of pain‐free parents (see Table 2). The covariate adolescent age made a significant contribution (*F*(1,48) = 8.45, *p* < 0.01, η_p_^2 ^= 0.15), indicating a stronger health‐threat‐related interpretation bias with increasing age. Results of the one‐sample *t* test within groups showed significant effect in both conditions, indicating that healthy offspring of both parents with chronic pain complaints and pain‐free parents are more likely to create non‐health‐related than health‐related words (offspring of parents with chronic pain complaints *t*(23) = −5.98, *p *< 0.001, 95% CI [−56.87 to −27.65]; offspring of pain‐free parents *t*(26) = −8.97, *p *< 0.001, 95% CI [−65.03 to −40.79]). Pearson correlations showed no significant associations between the interpretation bias index and parental pain catastrophizing (*r *= −0.13, *p *= 0.36), parental anxiety (*r *= −0.21, *p *= 0.13) and parental depression (*r* = 0.004, *p *= 0.98).

#### 3.2.3 Pain catastrophizing, anxiety sensitivity and somatisation

Results of ANCOVA’s on pain catastrophizing, anxiety sensitivity and somatic symptoms showed no differences between offspring of parents with pain complaints and offspring of pain‐free parents (see Table 3). The covariate age was not significant in all analyses (all *p *> 0.25). Pearson correlations showed no significant associations between parental pain catastrophizing or parental anxiety/depression and offspring's scores of pain catastrophizing, anxiety sensitivity and somatic symptoms (see Table 3).

**Table 3:** Mean scores and correlations between pain catastrophizing, anxiety, somatic symptoms and depression scores for adolescents and parents with and without chronic pain complaints

	Offspring of parents with chronic pain complaints (*n* = 24)		Offspring of pain‐free parents (*n* = 27)		*F*	*p*	η_p_^2^		Parent Pain catastrophizing	Parent Anxiety	Parent Depression
	*M* (*SD*)	Range	*M* (*SD*)	Range					Pearson *r* (*p*)		
Offspring pain catastrophizing	13.17 (7.30)	6–29	15.22 (8.03)	2–30	0.699	0.41	0.01		−0.014 (0.92)	0.14 (0.33)	0.09 (0.49)
Offspring anxiety sensitivity	26.96 (4.41)	21–34	26.78 (5.75)	18–43	0.062	0.81	0.00		0.088 (0.54)	0.17 (0.25)	0.09 (0.54)
Offspring somatic symptoms	16.29 (11.69)	0–45	16.41 (11.37)	3–42	0.000	0.98	0.00		0.021 (0.89)	0.03 (0.82)	−0.37 (0.80)

## 4 DISCUSSION

This study was primarily set up to examine the occurrence of pain‐related attentional and interpretation biases in pain‐free offspring of parents with and without chronic pain complaints. A recently proposed conceptual model of the developmental psychopathology of chronic pain includes cognitive and affective pain vulnerability factors as potential mechanisms underlying the transmission of pain from parents to offspring (Stone & Wilson, 2016). On the basis of this model, we hypothesized that offspring of parents with chronic pain complaints would demonstrate a more pronounced pain‐related attentional bias and health‐threatening interpretation bias as compared to offspring of pain‐free parents. Furthermore, we expected higher levels of pain catastrophizing, anxiety sensitivity and somatic symptoms in offspring of parents with chronic pain complaints. In contrast to our hypotheses, no differences between both offspring groups were observed on any of the cognitive and affective vulnerability measures. Adolescents in both groups were generally found to interpret ambiguous stimuli in a benign, non‐health‐threatening manner. Furthermore, results showed that adolescents of pain‐free parents showed a significant pain‐directed attention bias, while no such bias was observed in offspring of parents with chronic pain complaints.

Cognitive‐affective models of chronic pain (Eccleston & Crombez, 1999; Pincus & Morley, 2001) suggest that cognitive biases contribute to the onset and maintenance of chronic pain. Attentional and interpretation biases are proposed to increase fear and anxiety for pain, by increasing the salience of pain‐relevant stimuli or enhancing the threatening value of ambiguous stimuli. The increase in fear and anxiety will ensue a vicious cycle, in which these biases will be increasingly enhanced, triggering maladaptive behavioural responses to pain (e.g. avoidance). Meta‐analyses have offered support for this hypothesized role of cognitive biases in the pathogenesis of chronic pain, with patients being more vigilant towards pain‐related stimuli and more often endorsing a threat‐related interpretation of ambiguous stimuli as compared to healthy controls (Crombez et al., 2013; Schoth & Liossi, 2016; Schoth, Nunes, & Liossi, 2012).

Experimental studies on cognitive biases in children and adolescents with chronic pain are more sparse, and so far have yielded relatively consistent evidence for a biased threat interpretation (Brookes, Sharpe, & Kozlowska, 2018; Heathcote et al., 2017, 2016), but inconsistent or weak evidence for the presence of an attentional bias (Boyer et al., 2005; Brookes et al., 2018; Heathcote et al., 2018; van der Veek et al., 2014)*.* Lau and colleagues (2018) have argued that these mixed findings might be the result of a lack of ecological validity of the cognitive bias measures, in that they are unable to capture the fluctuating nature of biases in response to different situations and contexts. Likewise, it can be argued that cognitive biases have not stabilized yet in this specific age group, or simply have thus far not developed in these pain‐free adolescents of parents with chronic pain complaints. Although both attentional and interpretation biases have been suggested to be closely associated to one another (Todd et al., 2015), there is no consensus on the order and time course of their development.

Given that adult chronic pain patients are more vigilant towards pain‐related stimuli compared to healthy controls (Crombez et al., 2013; Pincus & Morley, 2001), we expected that this would also be the case for offspring of parents with chronic pain complaints who are more likely to be confronted with parental pain behaviours (Stone et al., 2018) and the consequence of pain than offspring of pain‐free parents. Our results unexpectedly showed that although the group difference was not significant, only adolescents of pain‐free parents showed a significant attention bias towards pain‐related stimuli. Given the inherent alarming function and aversive nature of pain (Eccleston & Crombez, 1999), the prioritization of pain‐related information in pain‐free adolescents is perhaps not that surprising and may even reflect a “healthy” response. In contrast, the offspring of parents with chronic pain complaints might have habituated towards pain stimuli, that is: being frequently exposed to various pain stimuli due to parental chronic pain may have reduced the alarming function of pain as often observed in healthy individuals (Eccleston & Crombez, 1999). This finding and proposed hypothesis needs to be further explored in future research.

The lack of an attention bias within offspring of parents with chronic pain complaints is in contrast with the literature concerning other psychological disorders (e.g., depression, anxiety disorders; e.g., Dearing & Gotlib, 2009; Muris et al., 2010). Note, however, that prior studies used different age groups compared to our study, with some including children that were younger (Dearing & Gotlib, 2009; Ellis et al., 2013; Kirsten et al., 2012; Lester, Field, Oliver, & Cartwright‐Hatton, 2009; Muris et al., 2010) and some including older adolescents (Forestell et al., 2012; Zetteler, Stollery, Weinstein, & Lingford‐Hughes, 2006). It is possible that cognitive biases develop in a fluctuating pattern making it more difficult to find a consistent pattern when comparing different age groups. Additionally, we aimed to identify pain‐related vulnerability within offspring of chronic pain patients and consequently recruited healthy, pain‐free adolescents for our study. However, by only including healthy adolescents, it is possible that the study suffered from a selection bias in that we mainly captured adolescents who were resilient to a chronic pain development. To be able to draw definitive conclusions about the intergenerational transmission of chronic pain and the role of cognitive pain‐related biases, we will need to conduct further research using longitudinal designs that follow children from a young age into adolescence when the prevalence of chronic pain increases (King et al., 2011; Perquin et al., 2000).

Some limitations of the current study should be noted. First, although the mean pain duration for parents was almost 14 years, the range was large, with some parents only having chronic pain complaints for 6 months. One could reason that this pain duration is not long enough to already elicit an attention bias within the healthy child. Alternatively, it can be suggested that not so much the mere presence of parental pain, but rather the manner in which parents cope with their pain complaints, and how they interact about their pain with their children are important for the development of vulnerabilities in the child (Cordts et al., 2019; Stone et al., 2018). In the current study, parental pain catastrophizing and parental psychological distress levels were not found to be related to pain‐directed cognitive bias in the child. However, it should be noted that parental distress levels were in the normative range, which limits the interpretability and generalization of the results. Future research should also incorporate other parent pain behaviours, like maladaptive coping behaviours or high levels of pain display to examine their effect on the presence of pain vulnerability, including pain‐directed cognitive bias, in the offspring. Likewise, it is recommended that future studies include additional information regarding parental pain characteristics (e.g. pain severity, pain frequency, pain intensity), the experienced level of disability when examining parental influences on offspring pain vulnerability. Second, we relied on self‐report rather than patient records to obtain information about chronic pain and psychopathology diagnosis. Moreover, by excluding parents with psychopathology, the current sample of parents with chronic pain complaints may have been overall healthier than the average chronic pain patient sample that commonly suffers from additional psychological disorders (Breivik et al., 2006; Chapman & Gavrin, 1999). Furthermore, we cannot rule out that the attentional bias results were influenced by factors such as lack of motivation or failure to follow the instructions to focus on the fixation cross, which is necessary to measure the shift of attention (Heathcote et al., 2015). Moreover, testing took place at home or at the university, which may have impacted task performance differently. Finally, notwithstanding the fact that studies with adult pain patients successfully used a similar word‐association approach to study interpretation bias (McKellar, Clark, & Shriner, 2003; Moss‐Morris & Petrie 2003), this is the first study that adopted this method in children and adolescents. Other tasks, which are less subject to literacy level and writing skills might be more appropriate to assess pain‐directed interpretation bias, especially in younger populations (e.g., (Heathcote et al., 2017; Heathcote et al., 2016).

Studying pain‐related cognitive biases in children and adolescents are especially important as these biases might still be amendable and responsive to preventive interventions (Heathcote et al., 2018; Lau, 2013; Lau et al., 2018). Empirical evidence on the development of these biases and how they relate to various pain‐related outcomes (i.e., pain severity, pain related fear or anxiety) could be used to develop or enhance treatment strategies for chronic pain in children and adolescents, which at this moment have at most modest effects (Cooper, Fisher, Anderson, et al., 2017a; Cooper, Fisher, Gray, et al., 2017b; Eccleston, Cooper, Fisher, Anderson, & Wilkinson, 2017; Fisher et al., 2014). Recent studies have yielded tentative support for the positive effects of cognitive bias modification as a stand‐alone or additive intervention to other interventions (i.e., CBT, treatment as usual) on anxiety symptoms in children and adolescents (Cristea, Mogoașe, David, & Cuijpers, 2015; Lau, 2013; Salemink, Wolters, & Haan, 2015).

In conclusion, the present study showed that offspring of parents with chronic pain did not differ from offspring of pain‐free parents regarding pain‐directed cognitive bias (attention and interpretation), pain catastrophizing, anxiety sensitivity or somatic symptoms. Further research is warranted to clarify the role of cognitive biases and other cognitive and affective factors in the intergenerational transmission of chronic pain. A better understanding of factors that underlie the transmission of the risk at chronic pain can identify important targets for the prevention and treatment of chronic pain in children.

## CONFLICT OF INTEREST

The authors declare no financial or other relationships that might lead to a conflict of interest.

## AUTHOR CONTRIBUTIONS

The authors L. Vancleef, M. Goossens and P. Muris contributed to the conception and design of the study. J. Boselie and L. Vancleef analysed the data. J. Boselie and L. Vancleef drafted the manuscript. All authors contributed to interpretation of the data, writing and revising the manuscript.

## ENDNOTE

1 One parent did not specify the exact pain duration but was still included in the parental pain group because the assessment interview revealed that the pain duration was >6 months.
