# Supplementary material for: The relation between parental chronic pain, pain‐related attention and interpretation biases in pain‐free adolescents
Source: Eur J Pain. 2019 Jul 15;23(9):1663–73. doi: 10.1002/ejp.1444 (PMC6790560; doi:10.1002/ejp.1444)
Supplement: Supplementary file 1 [file EJP-23-1663-s001.docx]

| **Table S1.** *Baseline characteristics of parents and adolescents, displayed per condition.* | | |
| --- | --- | --- |
|  | *Parent with chronic pain complaints*  *n=24* | *Pain-free parent*  *n=27* |
|  | *m (SD)* | *m (SD)* |
| Paid Employment parent (n, %) | 16 (66.7) | 18 (66.7) |
| Full-time | 4 (25.0) | 7 (38.9) |
| Part-time | 12 (75.0) | 11 (40.7) |
| Relationship status parent |  |  |
| Married | 21 (87.5) | 25 (92.6) |
| Living together | 3 (12.5) | 1 (3.7) |
| Single | - | 1 (3.7) |
| School absence adolescent through illness (n, %) |  |  |
| never | 14 (58.3) | 18 (66.7) |
| Less than 1 day/week | 7 (29.2) | 8 (29.6) |
| 2 days a week | 1 (4.2) | 1 (3.7) |
| 3 days a week | 1 (4.2) | - |
| other | 1 (4.2) | - |
